# Congenital Primary Essential Cutis Verticis Gyrata

**Published:** 2016-04-12

**Authors:** Andreas M. Lamelas, Peter J. Taub, Lester Silver, Aron Kressel

**Affiliations:** Mount Sinai School of Medicine, New York, NY

**Keywords:** cutis verticis gyrata, congenital, primary, essential, scalp

## DESCRIPTION

A 23-month-old Nigerian boy presented with 2, large soft-tissue folds extending from the forehead to the scalp longitudinally. The folds were present at birth and have continued to enlarge proportionally with the rest of the scalp. The patient has no other medical history.

## QUESTIONS

1. **What is cutis verticis gyrata?**
2. **What are the different classifications of this condition?**
3. **What is the treatment?**
4. **How is cutis verticis gyrata confirmed histologically?**

## DISCUSSION

Cutis verticis gyrata (CVG), coined by Unna in 1907, is a term used to describe a scalp condition characterized by convoluted folds and deep furrows that resemble the outer surface of the cerebrum.^1^^,^^2^ The first case was reported by Alibert in 1837 describing the characteristic cerebriform appearance of the scalp.^3^ Cutis verticis gyrata is a rare disorder affecting 1 of 100,000 males and 0.026 of 100,000 females in the general population.^4^

Initially classified as primary (idiopathic) or secondary CVG by Polan in 1955, CVG now falls under 3 categories: primary essential, primary nonessential, and secondary.^5^^,^^6^ In 1984, Garden distinguished primary essential from nonessential CVG as the absence of any neuropsychiatric pathology such as mental retardation, cerebral palsy, epilepsy, seizures, or ophthalmologic abnormalities.^5^^,^^7^ Secondary CVG occurs as a result of inflammatory processes such as eczema, psoriasis, folliculitis, impetigo, erysipelas, and pemphigus or other pathologies including hamartomas, nevi, acromegaly, and pachydermoperiostosis. Although secondary CVG can occur at any age with no apparent gender differences, primary CVG usually occurs postpubertal, with 90% of cases presenting before 30 years of age and rarely occurring in young children.^5^ Primary CVG usually presents as symmetric folds running anterior to posterior typically involving the vertex and the occiput. Secondary CVG commonly presents with variable folds, not running longitudinally.

Although primary CVG is a benign process, patients can undergo surgical treatment for cosmesis. Surgical approach is determined on the basis of the size and location of the folds. Treatment of larger folds may require a staged approach with tissue expansion and local flap reconstruction, or the surgeon may opt for partial resection of the most abundant section of the lesion.^4^ Smaller folds are usually amenable to local excision and primary closure with care taken to avoid distortion of the brow, eyelid, and hairline depending on the location of the folds.^3^

Histologically, primary CVG ranges from normal skin structure to thickened connective tissue with hypertrophy or hyperplasia of adnexal structures. Secondary CVG has variable histology based on the underlying cause.^4^

## SUMMARY

The patient present herein is unique because, unlike most cases of primary CVG, he presented with congenital asymmetric folds extending on to the forehead and has no associated medical problems. As discussed, most cases of primary CVG occur postpubertal, rarely occurring at birth. Only 10 cases of congenital primary CVG have been reported, of which 9 were reported in girls with Turner's syndrome and the other was of a female infant with a normal karyotype but severe developmental delay and hypotonia, making these all nonessential cases.^3^^,^^8^ This is the first documented case of congenital primary essential CVG in the literature. Because of the location and size of the folds, complete excision with primary closure was performed. To achieve an optimal closure, undermining in the subgaleal plane was done to maximize advancement of the adjacent soft tissue. Care was taken to reapproximate the hairline to avoid distortion, and multiple adjacent Z-plasties were created within the hair-bearing scalp to hide the future scar.

## Figures and Tables

**Figure 1:**
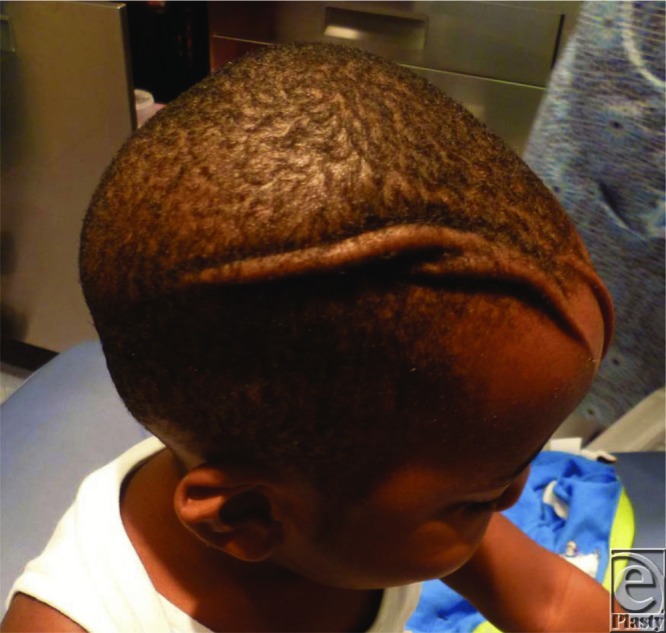
Patient at 12 months of age showing the 2 asymmetrical folds extending longitudinally from the forehead to the scalp.

**Figure 2:**
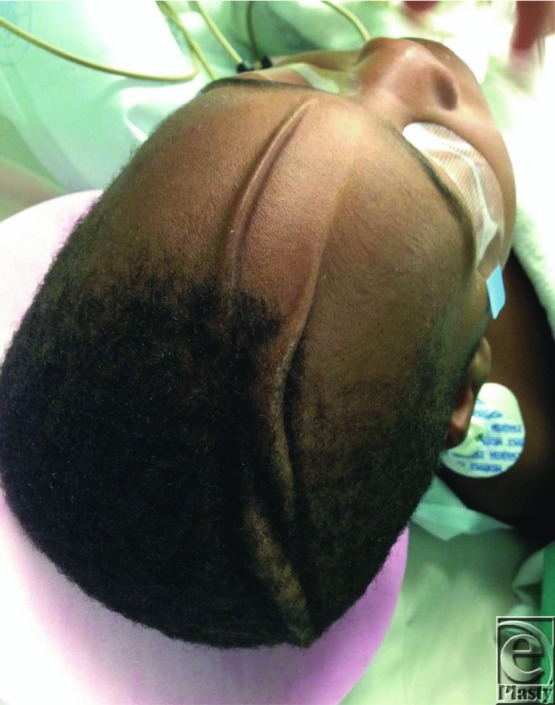
Patient at 23 months of age showing proportionate growth of the folds with the rest of the scalp.

**Figure 3:**
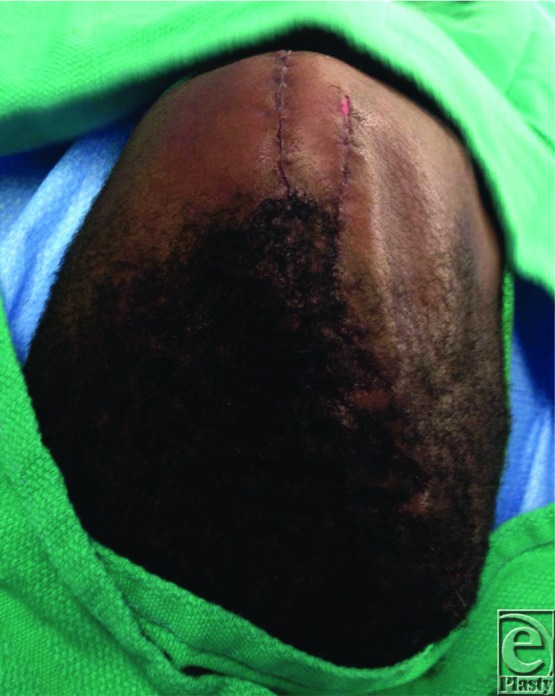
Intraoperative photograph of final wound closure showing multiple adjacent z-plasties within the hair-bearing scalp.

**Figure 4:**
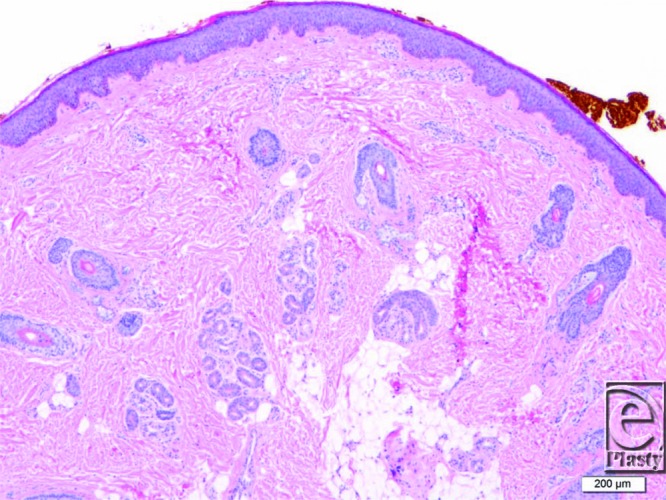
Histology slide showing an increase in collagen fibers with trapping of the apocrine and eccrine glands (original magnification ×20).
